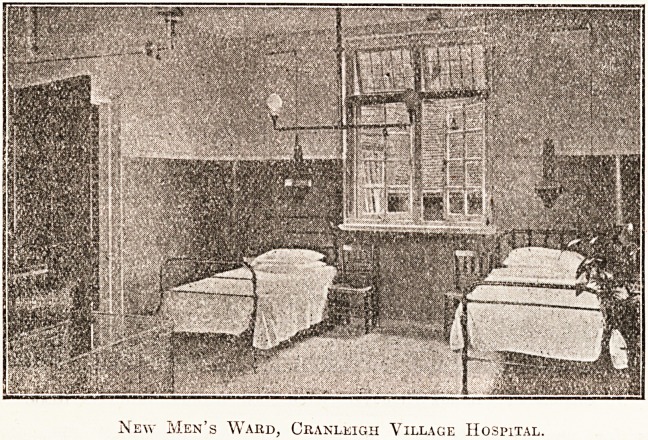# Reports on Hospitals of the United Kingdom

**Published:** 1914-03-14

**Authors:** Henry Burdett


### REPORTS ON
### Hospitals of the United Kingdom

By SIR HENRY BURDETT, K.C.B., K.C.V.O.

SERIES III.

### CRANLEIGH VILLAGE COTTAGE HOSPITAL.

Cranleigh Cottage Hospital was the first cottage hospital created and made successful among a relatively small village community by its founder, the late Mr. Albert Napper, F.R.C.S., who is known throughout the world as the father of the Cottage Hospital Movement. It must always have a historic and sentimental interest. The original building, which substantially remains, was a very old cottage of the ordinary Surrey type, given by the rector free of rent, and adapted by alterations and fittings for use as a hospital at a cost of about £50. The simple but effective manner in which alterations were carried out was worthy of all praise and displayed much ingenuity. The original building remains, and when we visited it on October 28, 1913, after an interval of quite twenty-five years, we were gratified to find that the managers have had the wisdom to preserve the original structure, and, by the removal of the old whitewash and paint, to expose the original oak beams and side pieces which have been polished. The old doors, too, probably of elm, have been treated in a similar manner, so that the original building as it can be seen to-day is in fact an interesting specimen of a cottage of the period to which it belongs. This work of preservation has been reverently done in fulfilment of the wish of the late Mr. Albert Napper, who desired that the first village hospital buildings should be permanently preserved as an instance of what could be done with very slight means. So Cranleigh Village Hospital is to-day distinguished as a landmark in the history of the cottage hospital movement.

Cranleigh Village Hospital, with New Wards Shown in Rear.

The frontispiece to the last edition of "Cottage Hospitals" (The Scientific Press) contains an elevation of the original building, and its picturesque character can be gathered from the illustration which we publish of Cranleigh Village Hospital, with the new wards shown in the rear. The buildings to-day comprise (1) the original cottage restored, which is at present occupied by the nurses and staff; and (2) the new buildings, consisting of the wards and operation theatre, &c., placed behind (1). A good idea of the nature of the reconstruction and the picturesque characteristics of the interior of the old buildings may be gathered from the illustration of one of the old wards, now a nurses' bedroom. No one who is interested in hospital work, or who is fond of seeing charming

One of the Old Wards, Cranleigh Village Hospital, now a Nurse's Bedroom.

buildings situated in some of the more beautiful portions of England's county gardens and scenery, should fail to pay a visit to Cranleigh, which is a village placed in one of the most picturesque portions of the county of Surrey.

One specially interesting feature is the linen-room, which is excellently planned and arranged in that portion of the old hospital which was formerly a bathroom, widely famed for its ingenious equipment. It is a matter of interest, and greatly to the credit of all concerned, that the smartness and thoroughness of the arrangements made for the linen at Cranleigh, which we visited on the afternoon of the day on which we had inspected the Guildford Hospital, compared very favourably with those at the larger institution.

In order that the hospital as it stands to-day may be appreciated on its merits it may be well to add that the new block of buildings at the rear of the hospital consists of two excellent wards, one for men and one for women, the character of which will be realised from the illustration given of the men's ward. There is also an operation room, simply but adequately equipped, which is well lighted and sufficient for its purpose. One of the features of the hospital to-day is the kitchen, placed between the new buildings and the old, which constitutes an attractive feature of the reconstruction scheme. Mr. A. Arthur Napper, a son of the founder, who has done good service as a member of the medical staff and general committee, in 1912 gave a piece of land, now included in the hospital site, which has rendered the new buildings efficient by securing an abundance of light and good ventilation for the men's ward and operation theatre.

New Men's Ward, Cranleigh Village Hospital.

We were much struck with the neatness, order, and smartness of the up-keep and management of every portion of the reconstructed hospital at Cranleigh, which reflect the greatest credit on the nursing staff, and especially on the Nurse-Matron, Sister Hyland, who is responsible for the management of the whole establishment. We did not have the pleasure of meeting the Honorary Secretary, Mrs. W. Welsh, but we had a very pleasant conversation with Mr. A. Arthur Napper, whom we were glad to see in robust health and full of energy and work.

Sixty-eight cases were under treatment during 1912, the last year for which a return is available. Of these fifty-eight were cured, three died, and seven were still under treatment. Sixteen operations were performed, and one patient was sent to St. Thomas's Hospital. The hospital contains eight beds, of which two are for emergency cases; the average number occupied is four. Small weekly payments are taken from the patients wherever possible, the receipts from this source having been £62 in 1912. The financial position of the hospital is satisfactory, and, after paying all outgoings for the year, the village hospital has a small but sufficient balance in hand. We are glad to note, too, that there is a balance of upwards of £100 to the credit of the Extension Fund. The report does not give the names of the honorary consulting staff. It includes, we believe, Mr. Napper and one or more surgeons of repute, one of whom, at least, is practising in London, but places his services freely at the disposal of the patients of this hospital whenever they may be required in a difficult case of diagnosis or for a specially severe and critical operation. It would rejoice, we are sure, the heart of the late Mr. Albert Napper, if he were able to go over the reconstructed village hospital at Cranleigh, to realise its many attractive features and the excellence of the work being done there in all departments.

## Figures and Tables

**Figure f1:**
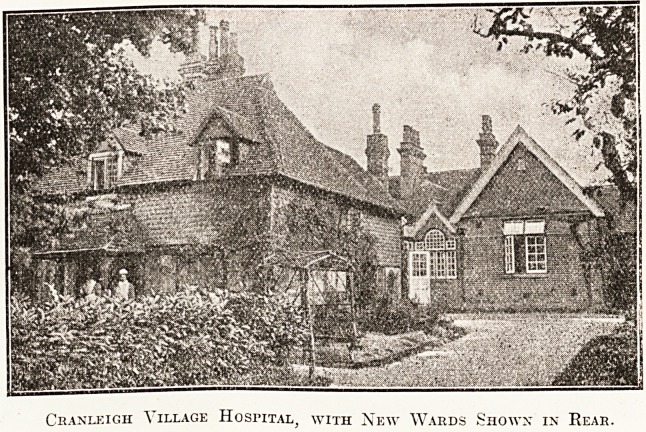


**Figure f2:**
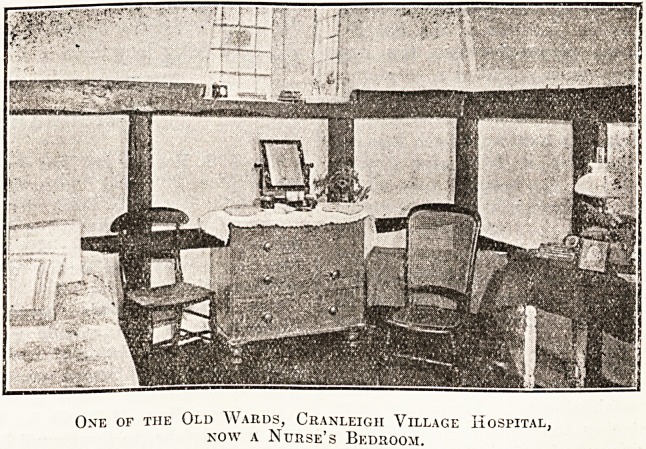


**Figure f3:**